# Ultrasound-Assisted Microencapsulation of Soybean Oil and Vitamin D Using Bare Glycogen Nanoparticles

**DOI:** 10.3390/molecules26175157

**Published:** 2021-08-25

**Authors:** Rita Cimino, Sukhvir Kaur Bhangu, Anshul Baral, Muthupandian Ashokkumar, Francesca Cavalieri

**Affiliations:** 1 Department of Chemical Sciences and Technology, University of Rome Tor Vergata, Via della Ricerca Scientifica 1, 00133 Rome, Italy; Rita.Cimino@uniroma2.it; 2 School of Science, RMIT University, Melbourne, VIC 3000, Australia; roop.bhangu@rmit.edu.au; 3 School of Chemistry, University of Melbourne, Melbourne, VIC 3010, Australia; anshulb@student.unimelb.edu.au

**Keywords:** glycogen, microcapsules, ultrasound

## Abstract

Ultrasonically synthesized core-shell microcapsules can be made of synthetic polymers or natural biopolymers, such as proteins and polysaccharides, and have found applications in food, drug delivery and cosmetics. This study reports on the ultrasonic synthesis of microcapsules using unmodified (natural) and biodegradable glycogen nanoparticles derived from various sources, such as rabbit and bovine liver, oyster and sweet corn, for the encapsulation of soybean oil and vitamin D. Depending on their source, glycogen nanoparticles exhibited differences in size and ‘bound’ proteins. We optimized various synthetic parameters, such as ultrasonic power, time and concentration of glycogens and the oil phase to obtain stable core-shell microcapsules. Particularly, under ultrasound-induced emulsification conditions (sonication time 45 s and sonication power 160 W), native glycogens formed microcapsules with diameter between 0.3 μm and 8 μm. It was found that the size of glycogen as well as the protein component play an important role in stabilizing the Pickering emulsion and the microcapsules shell. This study highlights that native glycogen nanoparticles without any further tedious chemical modification steps can be successfully used for the encapsulation of nutrients.

## 1. Introduction

Core-shell microcapsules with tailored structures and properties are of great interest due to their applications in different fields, such as food, pharmaceutical and cosmetics [1]. Typically, the microcapsules’ shell provides a physical barrier to protect the active agents confined in the core from degradation and oxidation as well as enable their controlled release [2,3]. Depending upon the preparation method of microcapsules, the drug release, the stability and the biological activity of the active agents can vary [4]. Ultrasound-assisted emulsification is one of most efficient techniques used for the preparation of microcapsules [5,6,7]. In this method, physical effects, such as strong shear forces and shockwaves generated by acoustic cavitation, induce the formation of a stable oil-in-water emulsion system [8,9]. It has been observed that ultrasonic homogenization can change the structural properties of biopolymers in aqueous phase, which may promote the adsorption of the biopolymer at the interface of oil-in-water, leading to stable micro- and nano-emulsions [9]. Different polysaccharides have been used to obtain stable Pickering emulsions, such as starch [10,11,12], modified starch [13], chitosan [14], cellulose [15], zein [16,17,18] and hydrophobically modified phytoglycogen [19,20]. In particular, it was reported that hydrophobically modified phytoglycogen increases the stability against coalescence and Ostwald ripening of oil-in-water Pickering emulsions [19,20,21].

**Figure 1:**
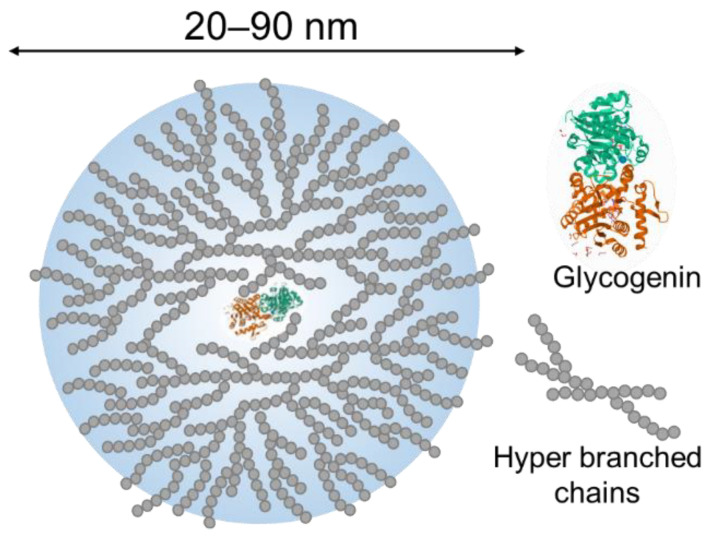
The structure of a glycogen nanoparticle showing the presence of glycogenin in the core and the hyper branched dendrimer-like architecture.

Glycogen is a biodegradable polymer nanoparticle used as a reservoir for glucose storage by animals and plants. It is a highly branched homo polysaccharide composed of repeating glucose units which are linked together linearly by α-d-(1→4) glycosidic linkage and branched by α-d-(1→6) glycosidic bonds, occurring every seven to fourteen glucose residues. Glycogenin is a protein that initiates the biosynthesis of glycogen and remain embedded in the core of the dendrimer-like nanoparticle (Figure 1) [22,23]. Glycogen can be extracted from oyster, bovine and rabbit liver and sweet corn and it is commercially available. Depending on the source, the molecular weight, size and structural properties of glycogens can vary, as summarized in Table 1. Glycogen has several advantageous properties, such as biocompatibility, biodegradability, high water solubility and hydroxyl functional groups, that make it well suited for use as a functional nanomaterial [24]. For instance, RNA and DNA delivery systems were engineered using bovine liver, oyster glycogen and phytoglycogen. Glycogen nanoparticles were functionalized with positively charged moieties to condense the negatively charge RNA or DNA molecules and facilitate their cellular internalization [25,26,27]. Furthermore, amphiphilic glycogen nanoparticles have been used as a vaccine adjuvant to potentiate the immune response against protein-based antigens [28,29]. Glycogen-Concanavalin A (Con-A) hollow microcapsules were developed by the layer-by-layer deposition technique exploiting the high affinity of Con-A to glycogen, for the controlled release of insulin [30,31]. These capsules were glucose responsive and tend to release the encapsulated insulin after incubation with glucose.

To the best of our knowledge, microcapsules made solely of unmodified/bare bovine liver, rabbit liver and oyster glycogen/phytoglycogen have not been reported. In this work, we applied the ultrasonic encapsulation technique to prepare soybean oil- and vitamin d-filled microcapsules composed of glycogen shells. We explored the ability of unmodified glycogen nanoparticles to form stable oil-filled microcapsules, without the use of any chemical functionalization or surfactant. We demonstrated that the protein component and the physicochemical properties of glycogen nanoparticles play a vital role in stabilizing the oil/water interface to ultimately obtain stable microcapsules.

## 2. Results and Discussion

### Preparation of Glycogen-Based Microcapsules

In this study, glycogen nanoparticles derived from oyster (OG), bovine liver (BG), rabbit liver (RG) and plants (PG) were used as building blocks to prepare oil-filled microcapsules by ultrasound-assisted Pickering emulsion formation. Pickering emulsion refers to an emulsion stabilized by particles and is applied in different field, such as pharmaceutical, cosmetic and food industries [33]. Compared to the conventional thermodynamically stable emulsions, Pickering emulsions do not require surfactants, as the droplet coalescence is inhibited by the adsorption of nanoparticles at the oil/water interface [34,35]. The surface activity, effective close packing and interactions between nanoparticles at the oil–liquid interface are important to control the size distribution and storage stability of the obtained oil-filled microcapsules. Depending on the source, the molecular weight, size and structural properties of glycogen nanoparticles vary. The nanoparticle size spans from approximately 20 to 90 nm with molecular weights ranging from the 0.4 × 10^6^ to 18 × 10^6^ Da [24]. A slightly negatively charged surface, due to the residual phosphate groups from the biological synthesis, and a small number of bound proteins were observed on glycogen nanoparticles (Figure 1, Table 1).

The protein content per mass of glycogen nanoparticle was found to be 8 μg/mg for OG, 5.3 μg/mg for BG, 2.9 μg/mg for RG and 1.6 μg/mg for PG [32]. Previous studies suggest that the protein component of glycogen may be either incorporated within the structure and/or situated on the surface of the particles [32,36]. We have recently shown that the protein component of OG can be exploited as an anchor point for the photopolymerization of poly(*N*-isopropylacrylamide) chains on the surface of glycogen nanoparticles [32].

Glycogen itself is a hydrophilic and highly hydrated polysaccharide [37]. However, we postulate that its surface activity may be affected by the size, rigidity and the bound proteins. The surface activities of the different glycogen nanoparticles suspensions—OG, RG, PG and BG were studied at water–air and water–oil interfaces. As reported in Table 1, the air/water interfacial tension of oyster glycogen solution was found to be 60.4 ± 2.7 mN/m (surface tension of water 72.8 mN/m at 20 °C), whereas the surface tensions of other glycogens with less protein content, i.e., RG, BG and PG, were ~72 mN/m, ~69 mN/m and ~68.2 mN/m, respectively. These results might suggest that the high protein content in oyster glycogen causes a decrease in interfacial tension. When the interfacial tension at soybean oil–water or soybean oil/aqueous solution of various glycogens was compared, a significant reduction from 24.5 ± 0.4 mN/m to ~12–14 ± 2 mN/m was found. These results indicate that glycogen nanoparticles can potentially stabilize an oil/water interface and be utilized for the preparation of stable oil-filled microcapsules.

**Scheme 1:**
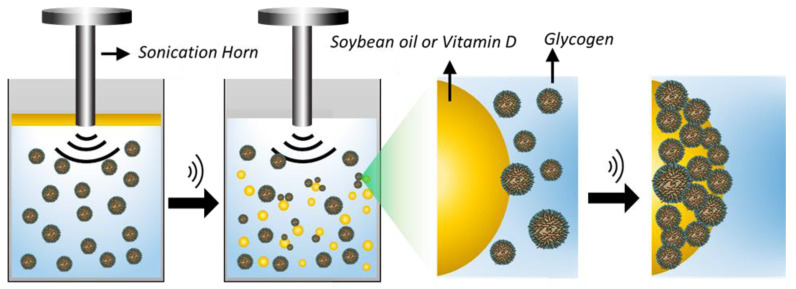
Illustration of the synthesis of glycogen microcapsules in aqueous solution using soybean oil as the organic phase.

**Figure 2:**
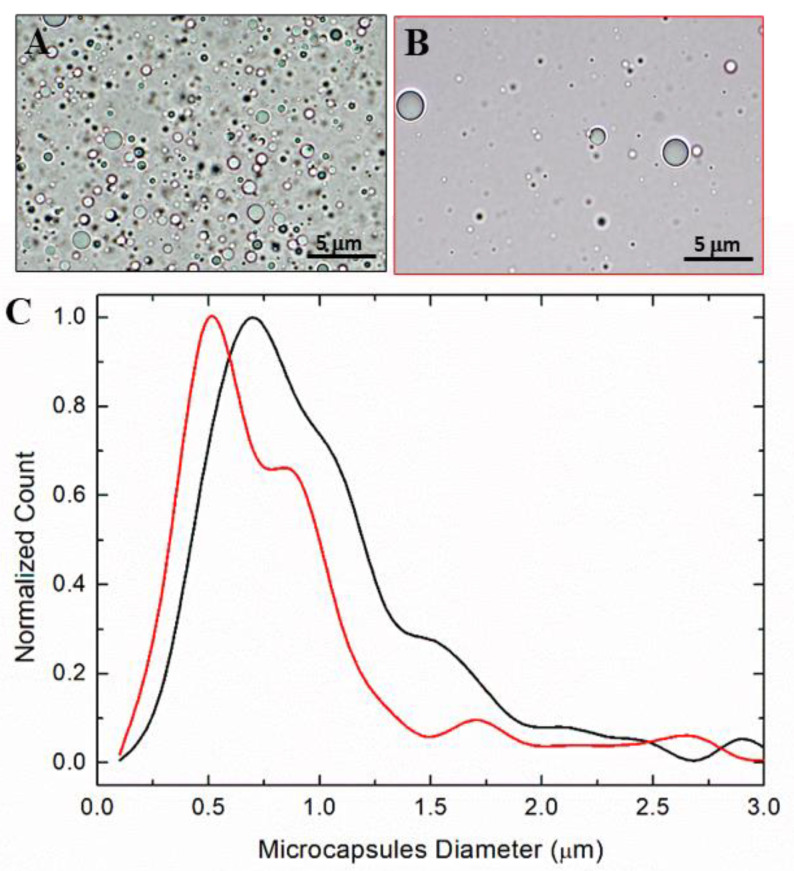
Optical microscopic images of RG_mc_ prepared using 10 mg/mL RG + 50 μL soybean oil (SO): (**A**) after preparation and (**B**) (red box) after one-month storage. (**C**) Comparison of normalized size distribution of RG_mc_ at day one (black) and after one month (red).

Next, glycogen-based microcapsules (mc) containing soybean oil were prepared by mixing 1 mL of glycogen aqueous solution with 50 µL of oil. High intensity ultrasound (20 kHz) was applied for 45 s at an applied acoustic power of 160 W by placing the tip of a 3 mm ultrasonic horn at the oil-water interface to obtain oil-filled glycogen microcapsules. A schematic illustration of the ultrasound induced encapsulation of soybean oil is provided in Scheme 1. The obtained microcapsules were washed twice by centrifugation and were analyzed by optical microscopy and dynamic light scattering, DLS. Firstly, glycogens from rabbit (RG) and bovine (BG) liver were used to synthesize microcapsules and their sizes and stability was compared over time. Figure 2 illustrates the size distribution and images acquired by optical microscope of the RG microcapsules (RG_mc_) obtained immediately after sonication and after one-month storage. Figure 2A,B show a dramatic decrease in concentration of RG_mc_ after one month (Figure 2B), indicating the limited stabilizing property of RG. Particularly, we observed the leaking of the oil phase over time and decrease in the average size of the capsules from 0.8 to 0.5 µm after one month (Figure 2C). This suggests that the larger capsules are less stable and disappear by coalescence to form an oil layer or can shrink due to oil leakage, leaving behind smaller capsules, whereas the smaller capsules remain stable over time.

**Figure 3:**
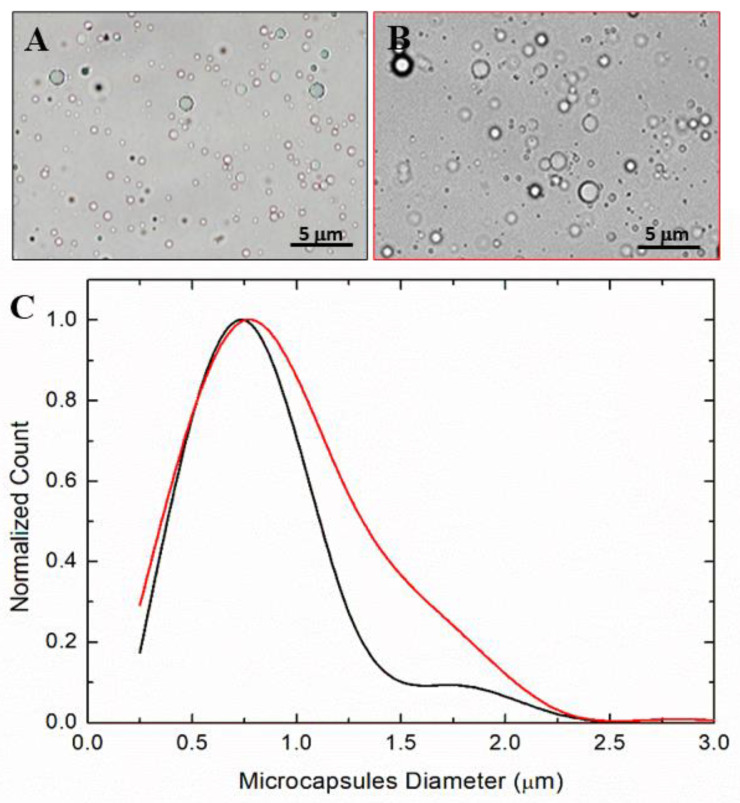
Optical microscopic images of glycogen from bovine liver microcapsules (BG_mc_) prepared using 20 mg/mL BG + 50 μL SO: (**A**) (black box) day one and (**B**) (red box) mc after one month. (**C**) Comparison of normalized size distribution of BG_mc_ day one (black) and one month later (red).

In contrast, it can be observed from optical microscopic (Figure 3A,B) images that there was no significant decrease in the concentration of the bovine glycogen microcapsules, BG_mc_, after one-month storage, although the presence of large microcapsules due to Ostwald ripening was observed. The diameter of fresh BG_mc_ ranges from 0.3 to 2.5 µm with the average value of ~0.7 µm. After one-month storage, a broadening of the DLS size distribution indicating the formation of larger microcapsules was noticed (Figure 3C), in agreement with the optical microscopy observation. Although the O/W surface tension and protein content of BG and RG are similar, these results indicate that the small BG nanoparticles can efficiently diffuse and interact at the oil/water interface to form a stabilizing shell, whereas the large RG nanoparticles exhibited very limited stabilizing properties. It should be noted that, due to the small size of BG, a higher concentration of BG (20 mg/mL) was required to form stable BG_mc._

**Figure 4:**
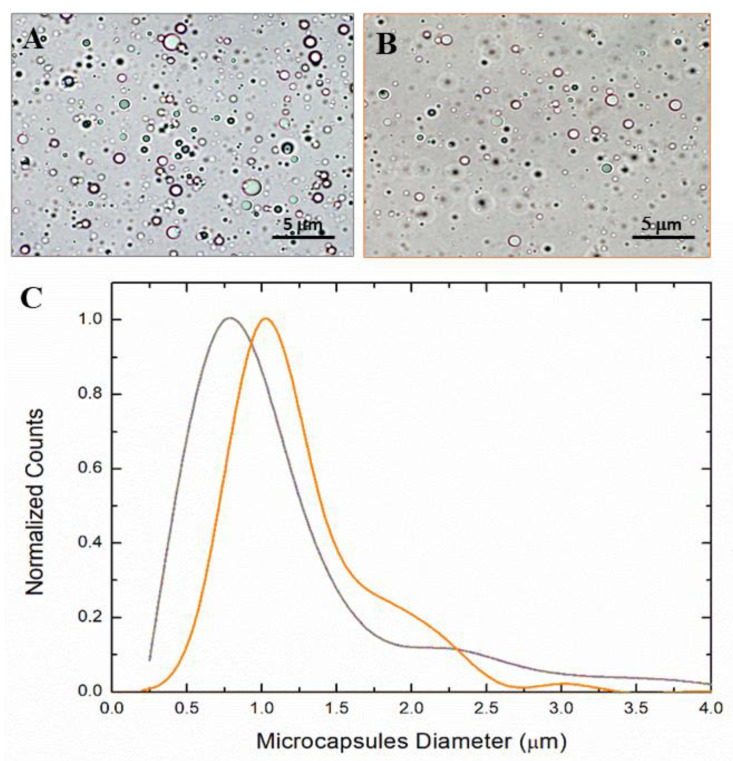
Optical microscopy images of oyster glycogen microcapsules (OG_mc_) prepared using 10 mg/mL with (**A**) 50 μL Soybean Oil (SO) and (**B**) 30 μL of SO. (**C**) Comparison of the normalized size distribution of OG_mc_ obtained by embedding 30 μL (orange) and 50 μL (grey) of SO.

Next, the formation of oyster glycogen microcapsules, OG_mc_ (OG has the highest protein content and size lying in between RG and BG), was investigated. The effect of various reaction parameters such as sonication time, power and concentration on the microcapsules properties was examined. Firstly, the effect of different amounts of soybean oil, i.e., 30 µL and 50 µL, at fixed oyster glycogen (10 mg/mL) concentration and sonication power (160 W) was tested. Figure 4A,B show the optical microscopic images of the OG_mc_ obtained with different soybean oil content. Both OG_mc_ preparations exhibited a bimodal distribution with similar mean diameters of 1.0 ± 0.2 µm and 1.2 ± 0.3 µm, respectively (Figure 4C). The fraction of OG_mc_ in the range 2–3 µm is likely formed through the coalescence of oil microdroplets induced by the shear stress generated by the ultrasonic treatment.

**Figure 5:**
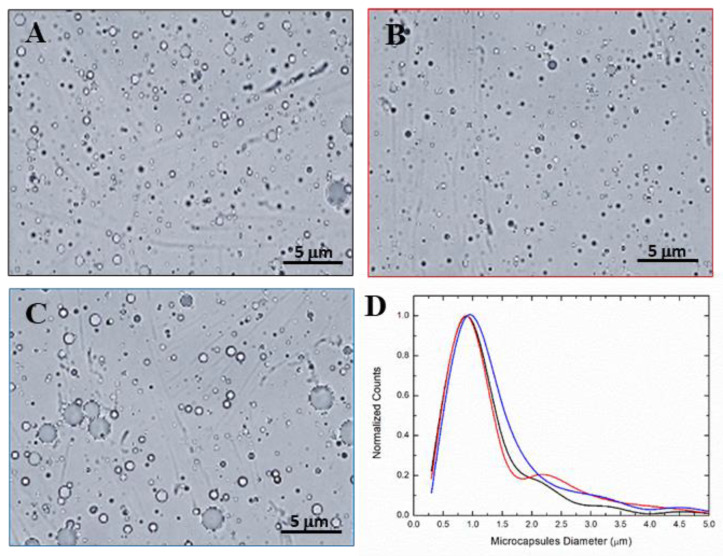
Optical microscopy images of OG_mc_, embedding 50 μL SO at different sonication times: (**A**) 30 s, (**B**) 45 s and (**C**) 60 s. (**D**) Comparison of the normalized size distribution of OG_mc_ obtained after 30 s (black line), 45 s (red line) and 60 s (blue line) of sonication.

The effect of the sonication time (three different times were chosen: 30, 45 and 60 s) on the size and size distribution of OG_mc_ was also evaluated (Figure 5). We found that the sonication time did not significantly affect the average size of the microcapsules (Figure 5A–D); however, the presence of large microcapsules with 60 s sonication time was observed due to coalescence (Figure 5C) and complete encapsulation of soybean oil was obtained only when the sonication was performed for 45 s. The size distributions of OG_mc_ obtained at different sonication times (Figure 5D) shows that the average diameter of the OG_mc_ was ~1 µm.

**Figure 6:**
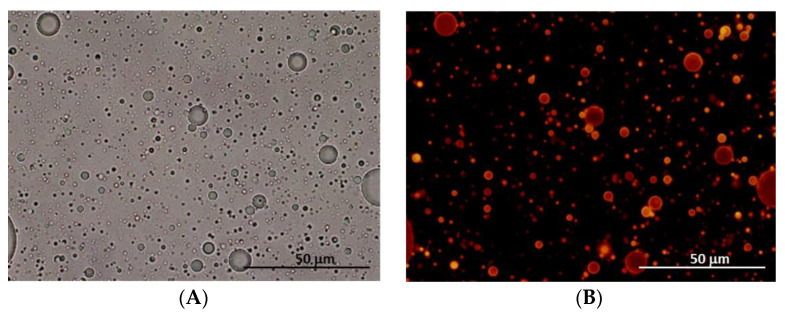
Optical (**A**) and fluorescence (**B**) microscopic images of soybean oil-filled microcapsules obtained using OG labeled with rhodamine (**B**) (acoustic power of 160 W and sonication time 45 s).

To further confirm the effective deposition of OG nanoparticles at the oil/water interface, the fluorescence probe rhodamine B was added in the microcapsules’ suspension. Figure 6 illustrates the fluorescence microscopy of the microcapsules exhibiting a higher red intensity at the interface due to the adsorption of the dye to the shell, confirming the formation of a compact stabilizing layer of OG nanoparticles (Figure 6). The microcapsules integrity was preserved even after high-speed centrifugation.

**Figure 7:**
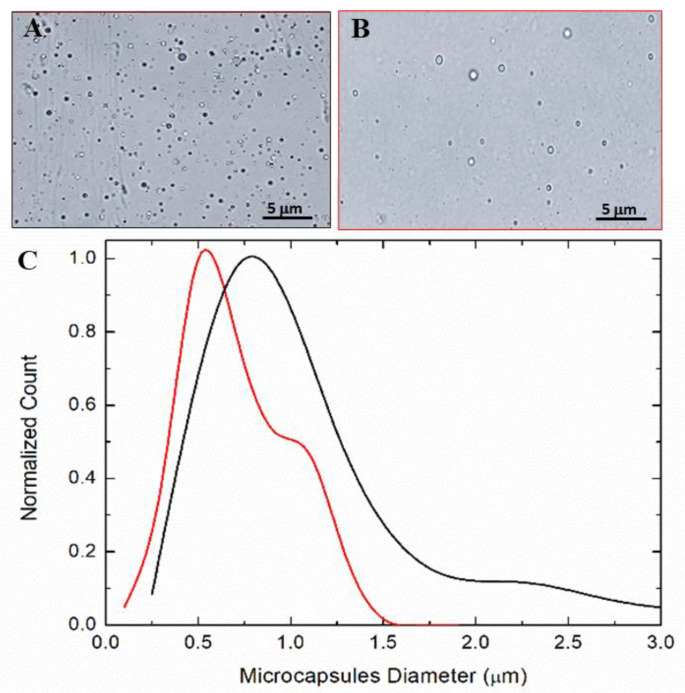
Optical microscopy images of OG_mc_ obtained using 10 mg/mL and 50 μL soybean oil: (**A**) immediately after preparation and (**B**) after one month. (**C**) Comparison between the normalized size distributions of OG_mc_ at day one (black) and one month later (red).

The stability of OG_mc_ was monitored up to one month. Figure 7 shows that the size of OG_mc_ decreased from 0.9 ± 0.3 µm to 0.7 ± 0.3 µm. This signifies that the larger microcapsules were not stable over prolonged period and were prone to disassemble and release the oil, which was also visually observed as a layer on top of the aqueous solution. Although the stabilizing effect of OG did not prevent the oil leakage from the larger microcapsules, the smaller microcapsules with a diameter ranging from 0.2 µm to 1.5 µm were found to be very stable. Overall, these data indicate that medium-sized OG nanoparticles can be effective in stabilizing oil-filled microcapsules. We can argue that the protein component of OG nanoparticles plays a vital role in preventing the coalescence of the small oil droplets, likely by gluing OG glycogen particles at the O/W interface via hydrophobic and hydrogen bond interactions.

We also explored the possible application of OG as an emulsifier for the encapsulation of lipophilic ingredients, which could be of great interest in the food and pharmaceutical industries [38]. As a proof of concept, we used vitamin D3, VitD, as an oily ingredient and OG as an emulsifier to form VitD-filled glycogen microcapsules.

**Figure 8:**
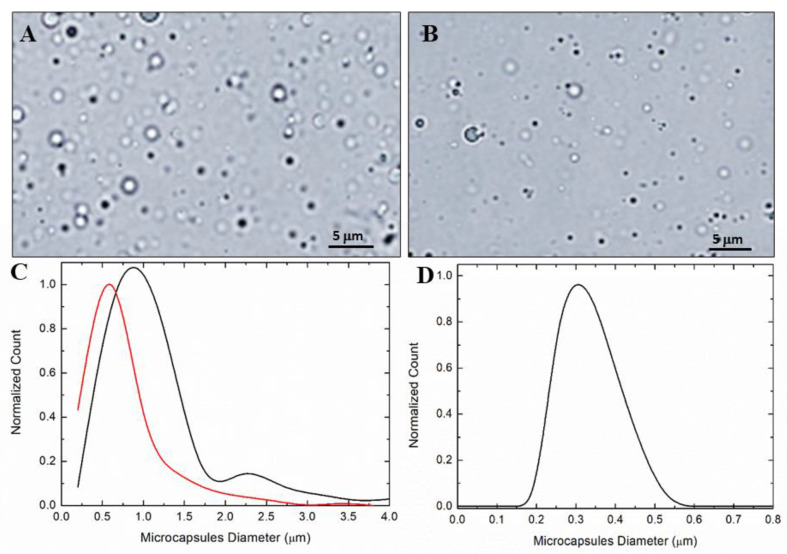
Optical microscopy images of day one (**A**) and after one month (**B**) of OG_mc_ prepared using 10 mg/mL OG + 50 μL VitD (**C**) Comparison between size distribution of OG_mc_ with VitD (red) and OG_mc_ prepared using 10 mg/mL OG + 50 μL SO. (**D**) Size distribution of OG_mc_ with VitD after one- month storage.

Vitamin D3 (VitD), also known as cholecalciferol, is easily degraded when subjected to light exposure or heat or in the presence of oxygen. Vitamin D deficiency is widely common in tropical countries because there are limited sources of vitamin D that can satisfy the required dietary allowance of this vitamin [39]. Therefore, microencapsulation of VitD is necessary to increase its bioavailability and stability, which may improve its persistence in processed foods. Comparing to soybean oil, which is a long-chain triglyceride oil, VitD is a seco-steroid molecule. The structure of the encapsulated agent [40,41] and its interfacial tension [42,43] can also affect the sizes and stability of microcapsules. The encapsulation of VitD was performed using OG and the procedure optimized for the encapsulation of soybean oil (10 mg/mL, 50 μL VitD, sonication time: 45 s, horn tip: 3 mm, acoustic power: 160 W). Figure 8 shows a comparison of the optical microscopy images and size distribution of OG_mc_, embedding soybean oil and VitD. It was evident that the VitD capsules have smaller size of 0.7 ± 0.2 µm and a narrower size distribution compared to the soybean oil capsules. After one-month storage, the diameter of OG_mc_ microcapsules with VitD was slightly reduced to 0.32 ± 0.08 µm (Figure 8D).

**Figure 9:**
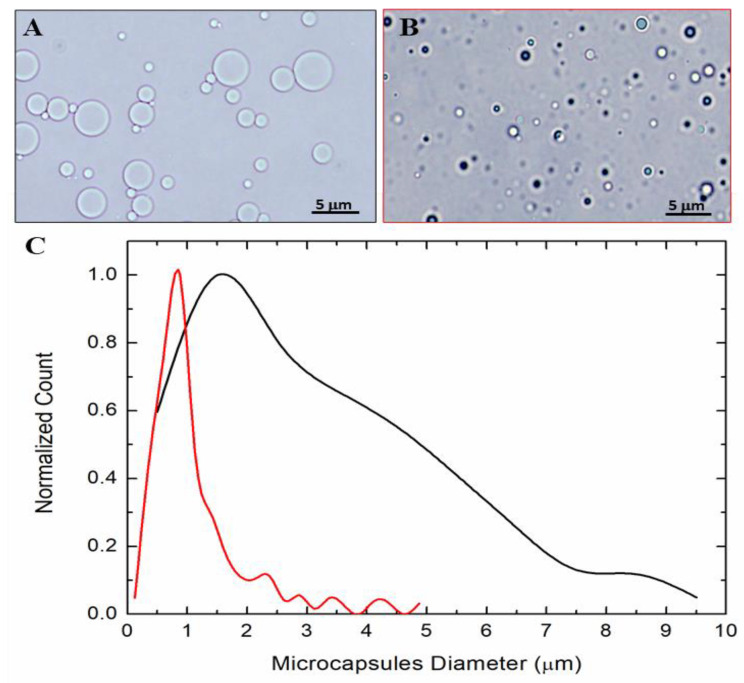
**(A**,**B**) Optical microscopy images of PG_mc_ embedding soybean oil and VitD, respectively. (**C**) Size distribution of PG_mc_ with soybean (black) and PG_mc_ with VitD (red).

**Figure 10:**
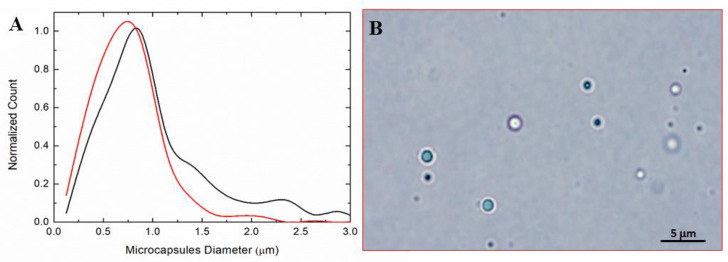
(**A**) Size distribution of PG_mc_ with VitD at day one (black) and after one month (red). (**B**) Optical microscopy image of PG_mc_ after one month.

Finally, phytoglycogen, PG, the largest glycogen nanoparticle with the lowest protein content (1.6 μg/mg), was tested for the encapsulation of both soybean oil and VitD. Interestingly, the ultrasonic encapsulation of soybean and VitD resulted in the formation of microcapsules with remarkable difference in size (Figure 9A,B). Large soybean oil-filled PG microcapsules were formed with a very broad size distribution and diameters ranging from 2 to 9 μm (Figure 9A). On the contrary, the VitD filled microcapsules appeared smaller with a narrow size distribution and an average size around 0.9 ± 0.3 µm (Figure 9C). PG_mc_-encapsulating soybean oil was not stable and released the oil after two weeks. PG_mc_-encapsulating VitD were monitored for one month, and it was found that along with a slight change in size, the yield was drastically reduced (Figure 10A,B).

Overall, there results indicate that phytoglycogen nanoparticles with a large size and a low protein content are not effective for the encapsulation of oily phases.

## 3. Materials and Methods

### 3.1. Materials

Oyster glycogen (OG), glycogen from bovine liver (BG), glycogen from rabbit liver (RG) and soybean oil (SO) were purchased from Sigma-Aldrich (Castle Hill, NSW, Australia). Phytoglycogen (PG) was purchased from Mirexus, Canada. Vitamin, D, as an oil-soluble nutrient from the brand Blackmore^®^ was purchased from a local pharmacy (Chemist Warehouse, Melbourne, Australia). All solutions were prepared with high purity water (Milli-Q) extracted from a Millipore system with resistivity of 18.2 MΩ/cm at 25 °C.

### 3.2. Synthesis of Microcapsules

Different glycogens were dissolved in Milli-Q at the concentration of 10 mg/mL or 20 mg/mL. Soybean oil (SO) and Vitamin D (VitD) were chosen for the encapsulation. SO- or VitD-filled glycogen microcapsules were obtained by layering 50 μL of the oil phase onto the glycogen solution (Table 2). The fluorescent dye Rhodamine B was added to the microcapsules’ solutions to label glycogen shells. A 20 kHz ultrasound horn (Branson Digital Sonifier) with a 3 mm diameter was placed at the oil-solution interface and the sonication was performed for 45 s at an acoustic power of 160 W. The obtained microcapsules were separated from the remaining solution by flotation and washed twice with Milli-Q water.

### 3.3. Optical and Fluorescence Microscopy

Fluorescence microscopy images of glycogen microcapsules were acquired using an inverted Olympus IX71wide field microscope with a 60× oil immersion objective equipped with a CCD camera (Cool SNAP FX, Photometrics, Tucson, AZ, USA). The specimen was irradiated with an illumination source with different wavelengths.

### 3.4. Dynamic Light Scattering (DLS), ζ-Potential

ZEN0040, Malvern Instruments (Malvern Panalytical Inc., Westborough, MA, USA) was utilized to determine the zeta potential and hydrodynamic diameter of the microcapsules. The measurement angle was 173° and the diameter of the particles was determined using the cumulant fit method. Microcapsules were dissolved in Milli-Q and in phosphate saline buffer 0.1 mM at pH 7.4 and zeta potential and hydrodynamic diameter of particles were determined.

### 3.5. Surface Tension Measurements

OG, RG, PG and BG were dissolved in MilliQ at the concentration of 10 mg/mL, 10 mg/mL, 10 mg/mL and 20 mg/mL, respectively. The surface tension was measured using the pendant drop method on OCA 15 EC in water/air and water/oil systems.

## 4. Conclusions

This study highlights a strong correlation between structural features of glycogens and their encapsulation properties. Large and low protein content nanoparticles, such as RG and PG, were found unable to effectively stabilize the O/W interface; therefore, they are not able to encapsulate the oily phases. In contrast, medium–small-sized and high protein content nanoparticles, such as OG and BG, were found to be suitable candidates for the encapsulation of oil phases. It can be concluded that a combination of the protein content and the particle size of glycogens plays a major role in the stability and size of the capsules. The rigidity and branching of glycogen nanoparticles may also be important factors to form a compact layer made of entangled polymer chains at the water oil interface, which may contribute to lowering the interfacial tension of the oil-filled microcapsules. The study also shows that OG can be used for the encapsulation of Vitamin D as an active ingredient and that the nature of the encapsulated ingredient can also alter the stability and size of the capsules. The Pickering emulsions prepared in this work are surfactant free and the results obtained by encapsulating bioactive ingredients with a biopolymer, such as glycogen, could be of great interest for the food and pharmaceutic industries.

## Figures and Tables

**Table 1:** Physicochemical properties of glycogen ^a^.

Sources of Glycogen	Oyster	Rabbit Liver	Bovine Liver	Phytoglycogen
Particle size (nm)	47 ± 16	80 ± 35	19 ± 4	88 ± 7
Molecular Weight (Da) ^a^	5.2 × 10^6^	8.6 × 10^6^	3.6 × 10^5^	1.8 × 10^7^
ζ-potential (mV)	0 ± 4	−2 ± 3	−11 ± 7	−1 ± 1
Protein content (μg/mg) ^b^	~8	~2.9	~5.3	~1.6
Surface tension (mN/m) in A/W	60.4 ± 2.7	71.9 ± 0.7	69.7 ± 0.4	68.2 ± 0.7
Surface tension (mN/m) in O/W	14 ± 2	12.0 ± 0.5	13 ± 1	13.4 ± 0.8
Degree of Branching (%) ^a^	5	5	9	3

**^a^** Data taken from Besford et al. [24]. **^b^** Data taken from Besford et al. [32].

**Table 2:** Experimental setup for the synthesis of microcapsules.

Sources of Glycogen	Concentration of Glycogen Solution (mg/mL)	Oil Phase	Sonication Time (s)	Acoustic Power (W)
Oyster	10	50 mL of SO 30 mL of SO 50 mL of VitD	60/45/30 45 45	160
Rabbit liver	10	50 mL of SO	45	160
Bovine liver	20	50 mL of SO	45	160
Phytoglycogen	10	50 mL of SO 50 mL of VitD	45 45	160

## Data Availability

Data supporting reported results can be found in internal repository set up at the University of Melbourne.

## Sample Availability

Samples of the compounds are available from the authors.
